# Supplementary material for: Pyrin dephosphorylation is sufficient to trigger inflammasome activation in familial Mediterranean fever patients
Source: EMBO Mol Med. 2019 Oct 7;11(11):e10547. doi: 10.15252/emmm.201910547 (PMC6835204; doi:10.15252/emmm.201910547)
Supplement: Supplementary file 1 — Appendix [file EMMM-11-e10547-s001.pdf]

Appendix

Table of contents:

Figure S1. **PKC inhibitors specifically trigger IL-1β release and a fast cell death in monocytes from FMF patients-genotypes indicated.** p2-6

Figure S2. **PKC inhibitors specifically trigger IL-1β release and cell death in monocytes from FMF patients.** p7-8

Figure S3. **Controls for the immunofluorescence staining presented in Fig 2A-B of the main manuscript.** p9

Figure S4. Staurosporine triggers Annexin-V$^+$/PI$^-$ and PI$^+$ cell death in monocytes from HD and FMF patients, respectively. p10

Figure S5. **Colchicine blocks inflammasome activation in monocytes from FMF patients following PKC inhibitor treatment (related to Fig 3 with genotypes indicated).** p11-12

Figure S6. Doxycycline-mediated expression of p.M694I, p.M680I and p.P369S Pyrin variants. p13

Figure S7. Edition of *PKN1/2* genes in U937 cells is associated with a strong bias towards indels not disrupting ORFs. p14

Figure S8. Validation of the anti-Phospho Pyrin antibody p15

Figure S9. Doxycycline-mediated expression of p.S208C, p.S242R, p.S208C/S242R, p.S208C/M694V, p.S242R/M694V, p.S208C/S242R/M694V Pyrin variants. p16

Figure S10. PKC inhibitors-mediated inflammasome activation specifically discriminates FMF patients from HD and from patients suffering from unrelated inflammatory conditions. p17

Appendix Table S1. Table of patients/ healthy donors (HD) p18-20

Appendix Table S2. Numerical parameters associated with the ROC curves presented in Fig. 6 p21

Appendix Table S3. Primer, sgRNA and siRNA table p22

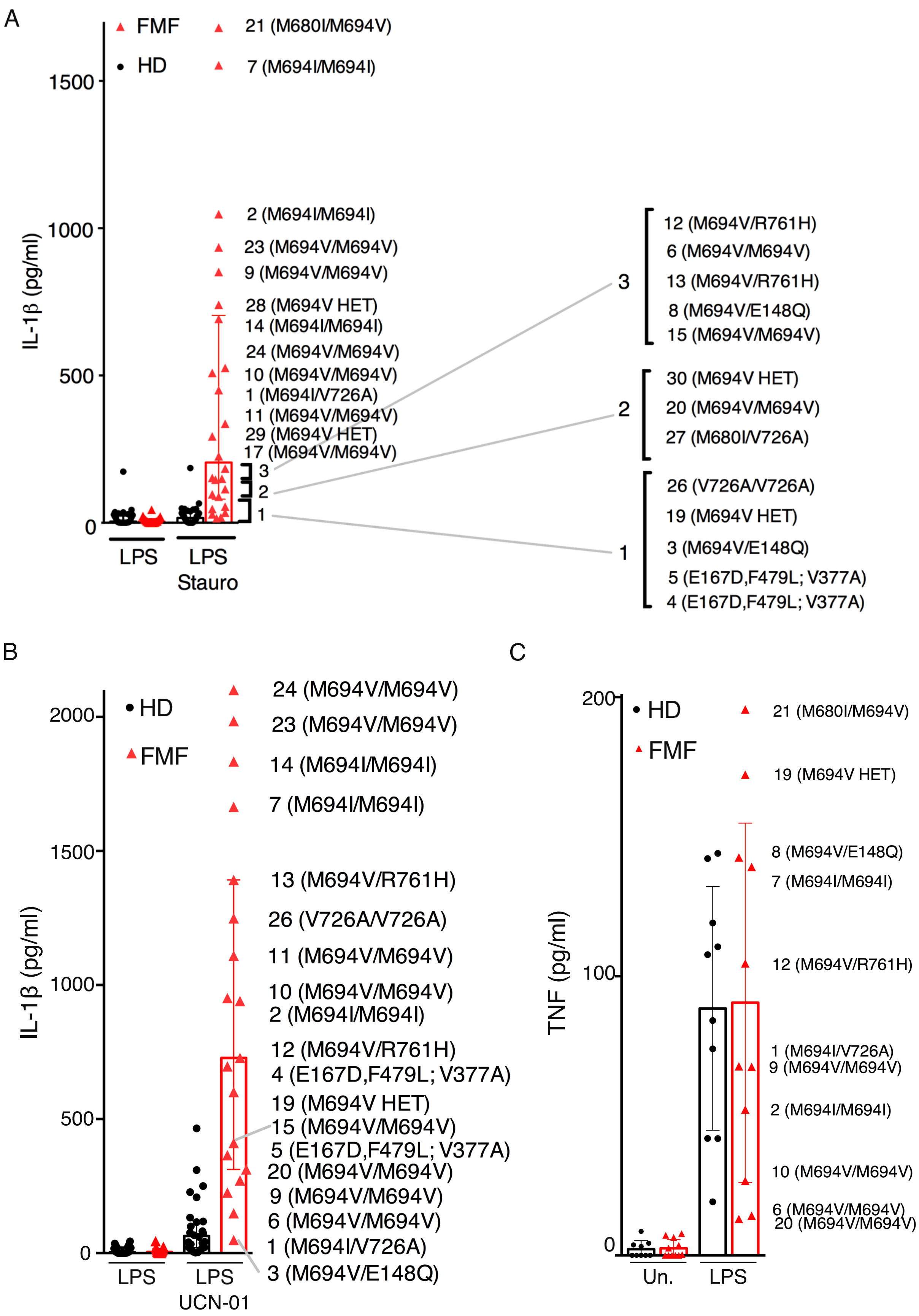
A
FMF
HD
21 (M680I/M694V)
7 (M694I/M694I)
IL-1β (pg/ml)
1500
1000
500
0
2 (M694I/M694I)
23 (M694V/M694V)
9 (M694V/M694V)
28 (M694V HET)
14 (M694I/M694I)
24 (M694V/M694V)
10 (M694V/M694V)
1 (M694I/V726A)
11 (M694V/M694V)
29 (M694V HET)
17 (M694V/M694V)
3
2
1
LPS
LPS Stauro
3
12 (M694V/R761H)
6 (M694V/M694V)
13 (M694V/R761H)
8 (M694V/E148Q)
15 (M694V/M694V)
2
30 (M694V HET)
20 (M694V/M694V)
27 (M680I/V726A)
1
26 (V726A/V726A)
19 (M694V HET)
3 (M694V/E148Q)
5 (E167D,F479L; V377A)
4 (E167D,F479L; V377A)
B
HD
FMF
IL-1β (pg/ml)
2000
1500
1000
500
0
24 (M694V/M694V)
23 (M694V/M694V)
14 (M694I/M694I)
7 (M694I/M694I)
13 (M694V/R761H)
26 (V726A/V726A)
11 (M694V/M694V)
10 (M694V/M694V)
2 (M694I/M694I)
12 (M694V/R761H)
4 (E167D,F479L; V377A)
19 (M694V HET)
15 (M694V/M694V)
5 (E167D,F479L; V377A)
20 (M694V/M694V)
9 (M694V/M694V)
6 (M694V/M694V)
1 (M694I/V726A)
3 (M694V/E148Q)
LPS
LPS UCN-01
C
HD
FMF
TNF (pg/ml)
200
100
0
21 (M680I/M694V)
19 (M694V HET)
8 (M694V/E148Q)
7 (M694I/M694I)
12 (M694V/R761H)
1 (M694I/V726A)
9 (M694V/M694V)
2 (M694I/M694I)
10 (M694V/M694V)
6 (M694V/M694V)
20 (M694V/M694V)
Un.
LPS

D

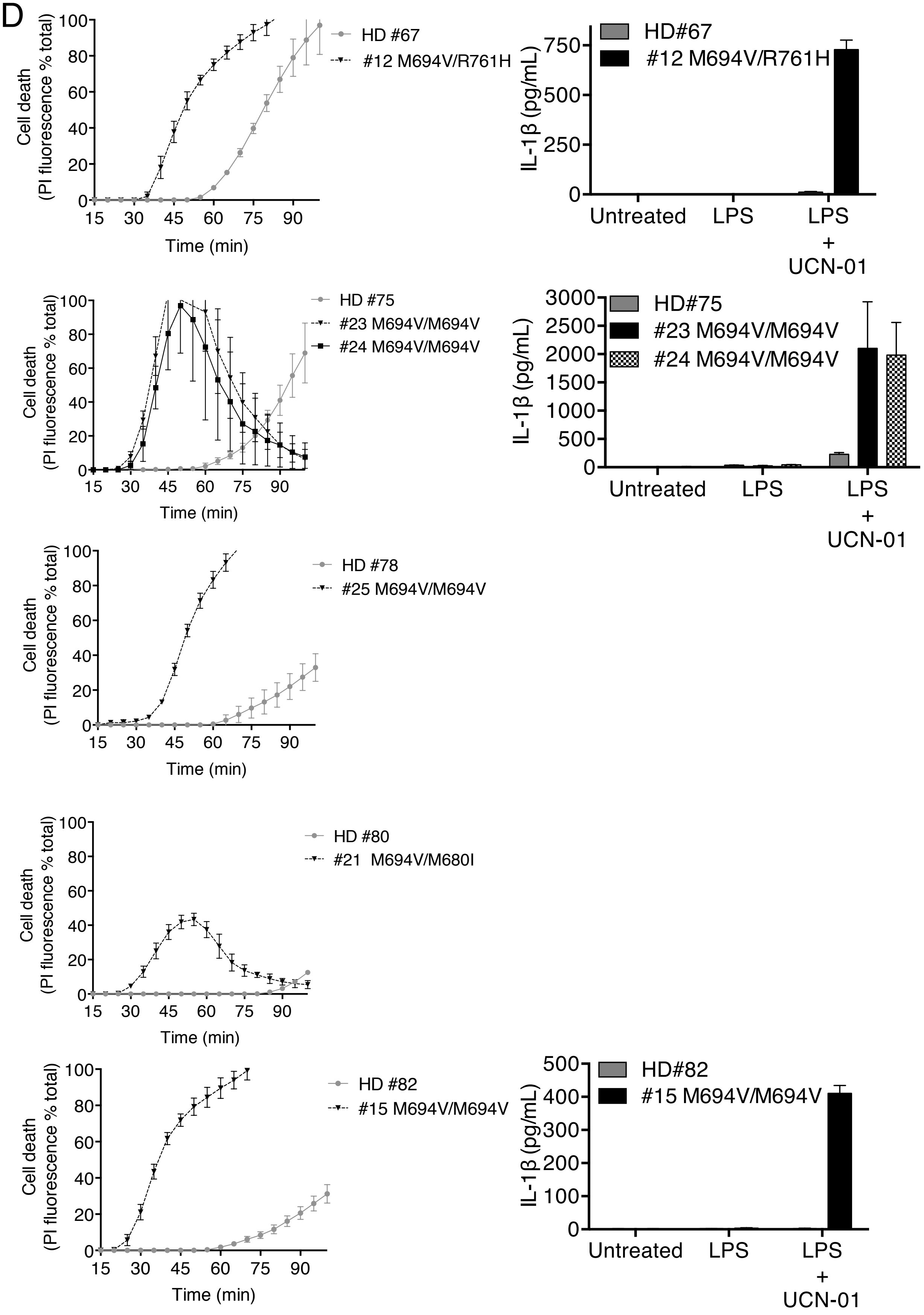

Cell death
(PI fluorescence % total)
Time (min)
HD #67
#12 M694V/R761H
IL-1β (pg/mL)
HD#67
#12 M694V/R761H
Untreated
LPS
LPS
+
UCN-01
HD #75
#23 M694V/M694V
#24 M694V/M694V
HD#75
#23 M694V/M694V
#24 M694V/M694V
HD #78
#25 M694V/M694V
HD #80
#21 M694V/M680I
HD #82
#15 M694V/M694V
HD#82
#15 M694V/M694V

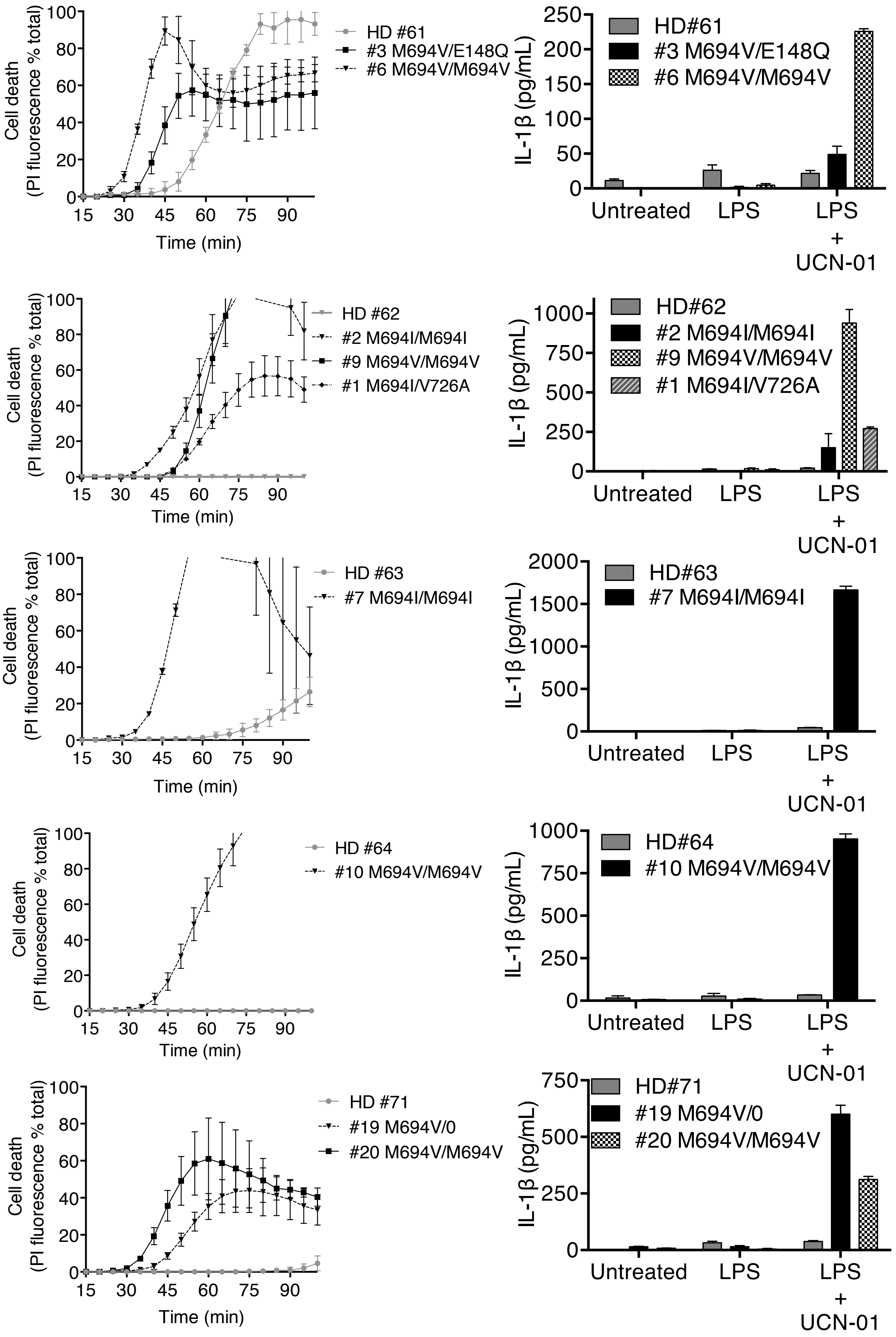
Cell death
(PI fluorescence % total)
0
20
40
60
80
100
15
30
45
60
75
90
Time (min)
HD #61
#3 M694V/E148Q
#6 M694V/M694V
IL-1β (pg/mL)
0
50
100
150
200
250
HD#61
#3 M694V/E148Q
#6 M694V/M694V
Untreated
LPS
LPS
+
UCN-01
HD #62
#2 M694I/M694I
#9 M694V/M694V
#1 M694I/V726A
0
250
500
750
1000
HD#62
#2 M694I/M694I
#9 M694V/M694V
#1 M694I/V726A
HD #63
#7 M694I/M694I
0
500
1000
1500
2000
HD#63
#7 M694I/M694I
HD #64
#10 M694V/M694V
HD#64
#10 M694V/M694V
HD #71
#19 M694V/0
#20 M694V/M694V
0
250
500
750
HD#71
#19 M694V/0
#20 M694V/M694V

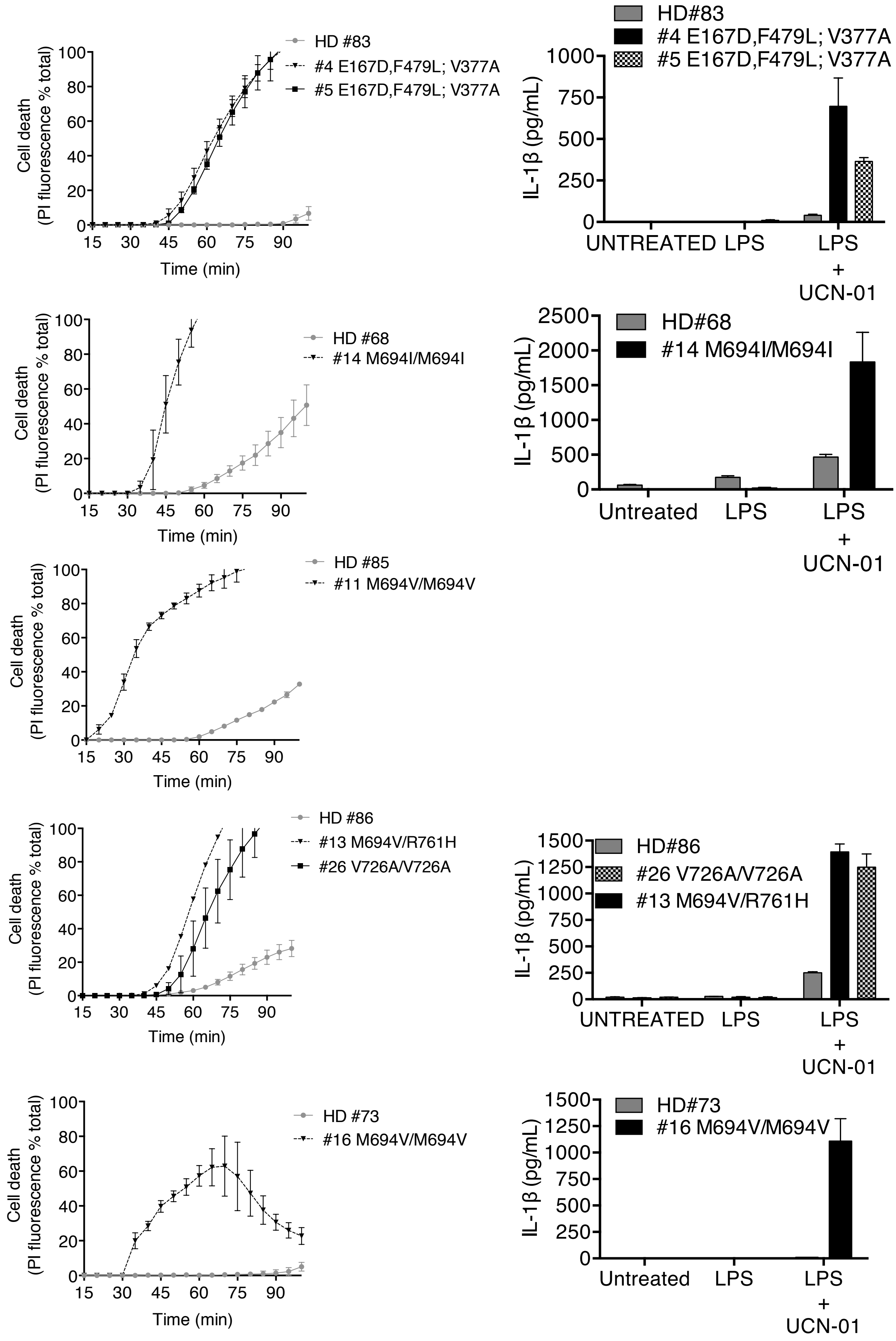
HD #83
#4 E167D,F479L; V377A
#5 E167D,F479L; V377A
Cell death (PI fluorescence % total)
Time (min)
HD#83
#4 E167D,F479L; V377A
#5 E167D,F479L; V377A
IL-1β (pg/mL)
UNTREATED
LPS
LPS + UCN-01
HD #68
#14 M694I/M694I
HD#68
#14 M694I/M694I
Untreated
HD #85
#11 M694V/M694V
HD #86
#13 M694V/R761H
#26 V726A/V726A
HD#86
#26 V726A/V726A
#13 M694V/R761H
HD #73
#16 M694V/M694V
HD#73
#16 M694V/M694V

Figure S1. PKC inhibitors specifically trigger IL-1β release and a fast cell death in monocytes from FMF patients-genotypes indicated.

Fig S1A, B, C correspond to Fig 1A, 1B, 1C of the main manuscript, except that they were expanded to present the genotype of each FMF patient. The indicated number corresponds to the patient number as indicated in Appendix Table S1.

FigS1D shows individual real time cell death assays and the corresponding IL-1β ELISA, when applicable.

Monocytes from healthy donors (HD) or FMF patients were either primed with LPS (A-C, D-right panels) or not (D-left panels) and treated with (A) 1.25 μM staurosporine (Stauro), (B, D) 12.5 μM UCN-01.

(**A**, **B**) IL-1β and (**C**) TNF level were quantified by ELISA at 1h30 post stimulation.

(**D**) Cell death was monitored in real time by measuring propidium iodide influx/fluorescence every 5 minutes.

Data information:

(A-C) Each dot represents the mean value from three technical replicates for one HD or patient. The bar represents the median ± interquartile range.

(D-left panels) Each point of the curve corresponds to the mean of a biological triplicate assessing cell death of one HD or one FMF patient.

(D-right panels) The bar represents the mean ±SD of a biological triplicate.

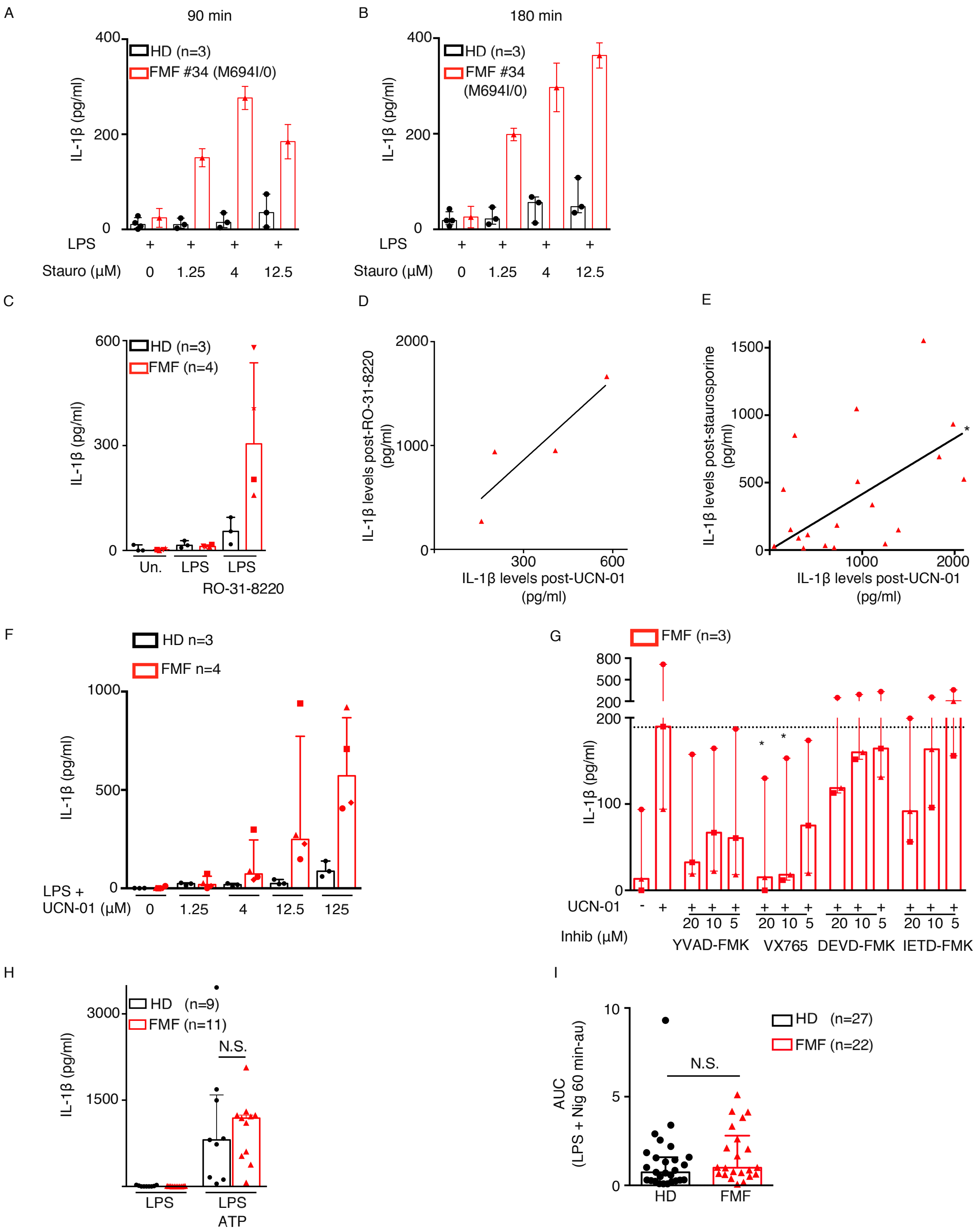

Figure S2. **PKC inhibitors specifically trigger IL-1β release and cell death in monocytes from FMF patients.**

Monocytes from healthy donors (HD) or FMF patients were primed with LPS and treated with (A, B) the indicated concentration of staurosporine for the indicated time; (C, D) 100 µM RO 31-8220; (D, E, G) 12.5 µM UCN-01; (E) 1.25 µM staurosporine; (F) the indicated concentrations of UCN-01, (H) 2.5mM ATP or (I) 50 µg/ml nigericin (nig), (A, C-H) for 1 h 30 or (B) for 3 h. (G) Caspase inhibitors were included at 30 min before UCN-01 at the indicated concentrations. The horizontal dotted line corresponds to the median value in the absence of inhibitor. (I) The area under the curve (AUC) corresponding to the curves presented in Fig. 1G were calculated from t=15 min to t-60 min to match the data presented in Fig. 1F. Due to the low level of cell death at 60 min post-nigericin, the area under the curve presented in Fig. 1I were calculated from t-15 min to t-105 post-nigericin.

Data information

(A-I) Each symbol represents the mean value from three technical replicates for one HD or FMF patient. (A-C, F-I) The bar represents the median ± interquartile range (except (A-B) in which a single FMF patient was included, the FMF bar represents the mean of a biological triplicate). (C) Symbol to patient #: square #2 (M694I/M694I), triangle #9 (M694V/M694V), triangle pointing down #7 (M694I/M694I), star #10 (M694V/M694V). (E) Correlation significance was determined by Spearman test, *: $p<0.05$. (F) Symbol to patient #: round #1 (M694I/V726A), square #2 (M694I/M694I), triangle #9 (M694V/M694V), diamond #6 (M694V/M694V). (G) Symbol to patient #: round #13 (M694V/R761H), square #39 (M680I/M680I), triangle #26 (V726A/V726A). Friedman test with Dunn's correction for multiple comparisons was performed, adjusted p-values are shown. VX765 20 µM *p=0.014; 10 µM *p=0.04. (H) N.S. p=0.77 by Wilcoxon rank-sum test. (I) N.S. p=0.16 by Wilcoxon rank-sum test.

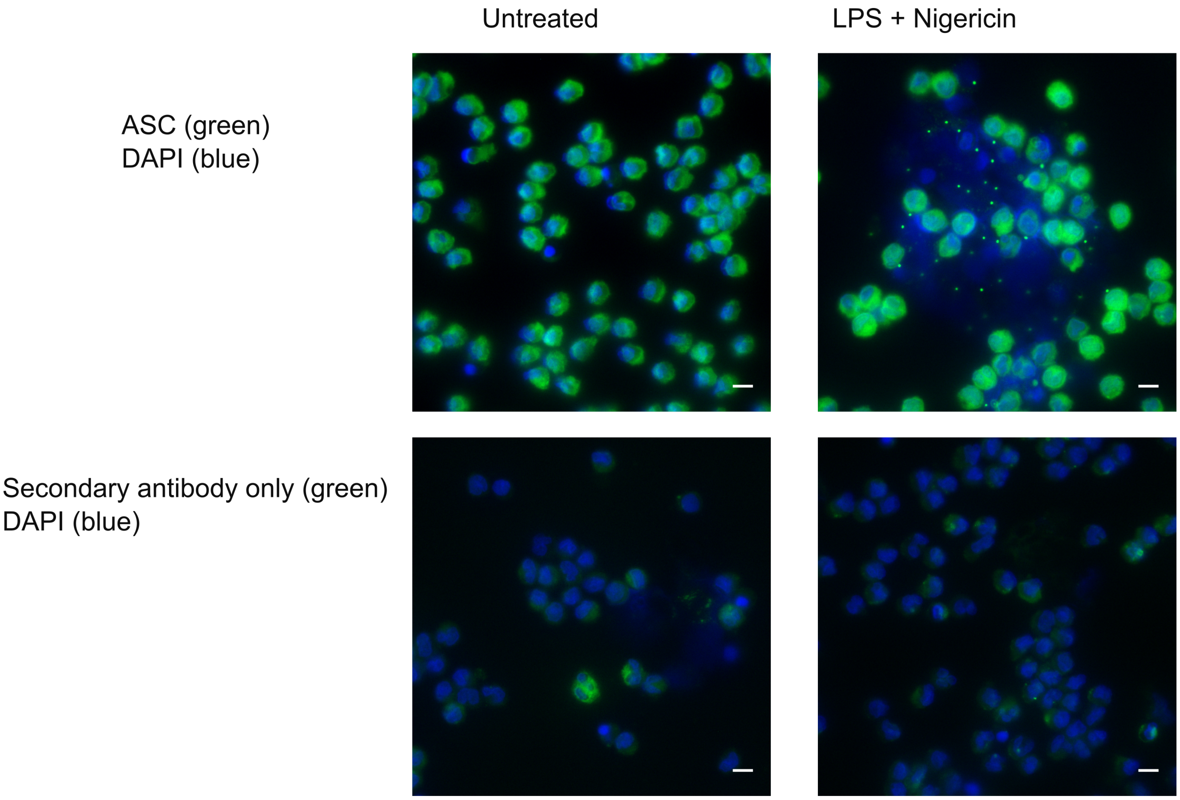

Figure S3. **Controls for the immunofluorescence staining presented in Fig 2A-B of the main manuscript.**

Monocytes from one healthy donor (HD) were left untreated (left panels) or treated with LPS (3h, 100 ng/ml) plus 5 μM Nigericin for 90 min. Cells in the top panels were stained with primary antibody against ASC (and Alexa-488-coupled secondary antibody-green) and DAPI, while cells in the lower panels were stained only with the secondary antibody. Representative images are shown. Scale bar 10 μm.

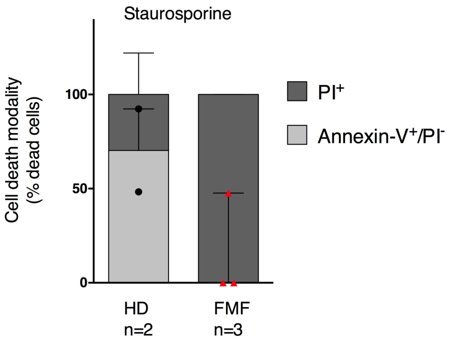

Figure S4. Staurosporine triggers Annexin-V^{+}/PI^{-} and PI^{+} cell death in monocytes from HD and FMF patients, respectively.

Cell death was assessed at 40 minutes post staurosporine addition by determining the percentage of treatment-induced Annexin-V^{+} PI^{-} cells and of PI^{+} cells among dead cells (Annexin-V^{+} and/or PI $^{+}$ cells) using flow cytometry.

Data information

Each dot (HD)/triangle (FMF) represents the value for one individual, the bar represents the median (+/- interquartile range).

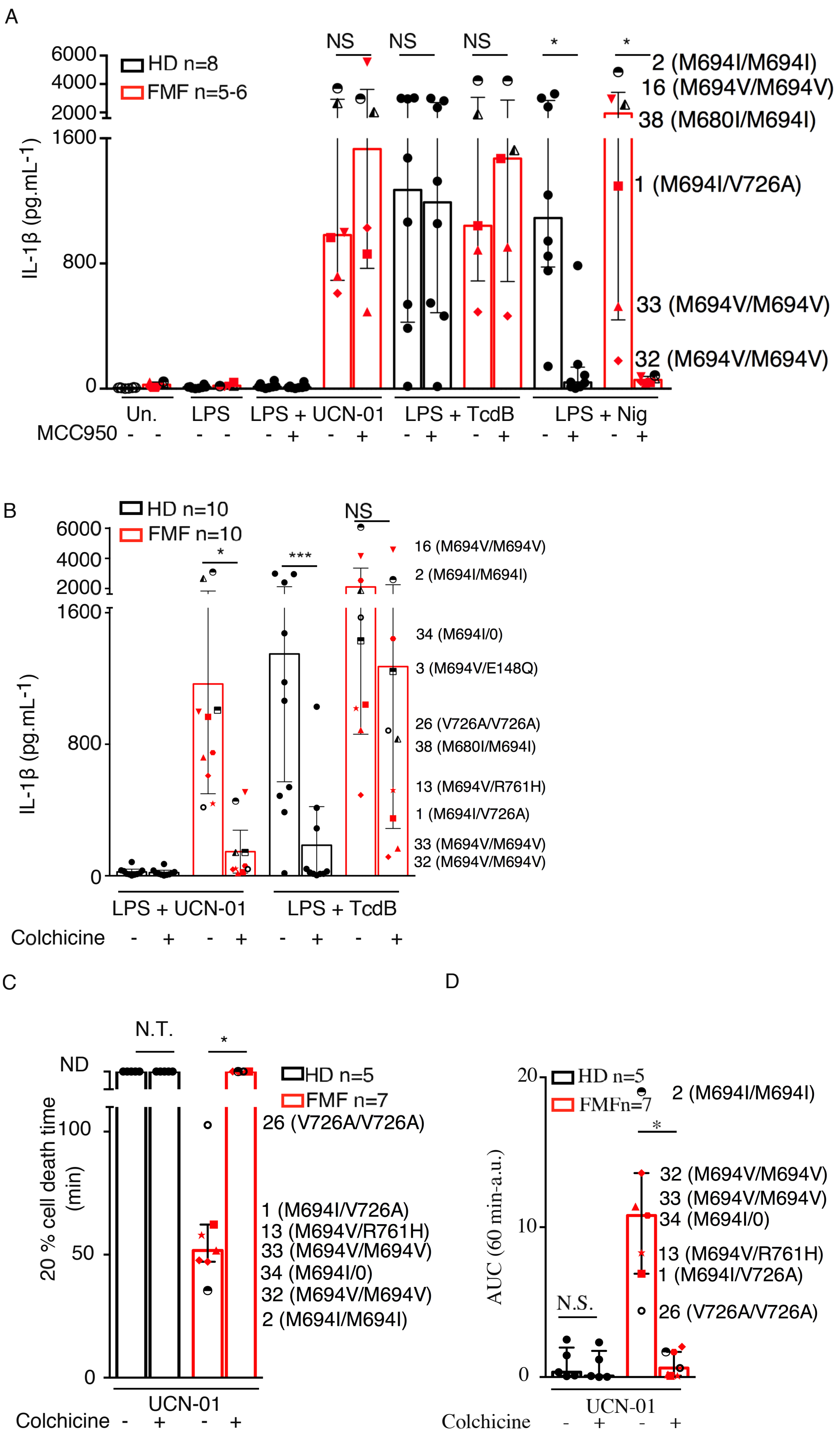
A
IL-1β (pg.mL-1)
HD n=8
FMF n=5-6
NS
NS
NS
*
*
2 (M694I/M694I)
16 (M694V/M694V)
38 (M680I/M694I)
1 (M694I/V726A)
33 (M694V/M694V)
32 (M694V/M694V)
Un.
LPS
LPS + UCN-01
LPS + TcdB
LPS + Nig
MCC950
B
IL-1β (pg.mL-1)
HD n=10
FMF n=10
NS
*
***
16 (M694V/M694V)
2 (M694I/M694I)
34 (M694I/0)
3 (M694V/E148Q)
26 (V726A/V726A)
38 (M680I/M694I)
13 (M694V/R761H)
1 (M694I/V726A)
33 (M694V/M694V)
32 (M694V/M694V)
LPS + UCN-01
LPS + TcdB
Colchicine
C
20 % cell death time (min)
N.T.
*
ND
HD n=5
FMF n=7
26 (V726A/V726A)
1 (M694I/V726A)
13 (M694V/R761H)
33 (M694V/M694V)
34 (M694I/0)
32 (M694V/M694V)
2 (M694I/M694I)
UCN-01
Colchicine
D
AUC (60 min-a.u.)
HD n=5
FMFn=7
2 (M694I/M694I)
*
32 (M694V/M694V)
33 (M694V/M694V)
34 (M694I/0)
13 (M694V/R761H)
1 (M694I/V726A)
26 (V726A/V726A)
N.S.
UCN-01
Colchicine

Figure S5. Colchicine blocks inflammasome activation in monocytes from FMF patients following PKC inhibitor treatment (related to Fig 3 with genotypes indicated).

Fig S4A-D correspond to Fig 3A-B, D-E of main manuscript, except that they were expanded to present the genotype of each FMF patient. The indicated number corresponds to the patient number as indicated in Appendix Table S1.

Monocytes from healthy donors (HD) or FMF patients were primed for 3 h as indicated with LPS (**A-B**) or not (**D-E**). When indicated, (**A**) the NLRP3 inhibitor MCC950 (10 μM) or (**B-D**) colchicine (1 μM) were added 30 min before addition of 12.5 μM UCN-01, 125 ng/ml TcdB or 5 μM Nigericin.

(**A**, **B**) IL-1β level was quantified by ELISA at 1 h 30 post stimulation.

(**C-D**) Cell death was monitored in real time by measuring propidium iodide influx/fluorescence every 5 minutes.

Data information

(**A, B, C, D**) Each symbol represents the mean value from three biological replicates for one HD or patient. The bar represents the median value (+/- interquartile range). (**C**) and the area under the curve (AUC) (**D**) were computed for each HD or FMF patients. Each symbol represents the mean value from one HD or patient. The bar represents the median (+/- interquartile range). (**C**) ND indicates that the average cell death was below 20% at the end of the kinetics. N.T.: Not tested.

(A) Wilcoxon matched-pairs signed rank test was used to compare untreated and MCC950-treated groups. FMF, UCN-01 NS : p=0.84, HD TcdB NS: p=0.81, FMF TcdB NS: p=0.84, HD Nig *p=0.039, FMF Nig *p=0.031. (B) Friedman paired test with Dunn's correction for multiple comparisons was applied to compare untreated and colchicine-treated groups. Adjusted p-values are as follow: FMF UCN-01 *p=0.036; HD TcdB ***p=0.0006; FMF TcdB NS p=0.28. (C-D) Wilcoxon matched-pairs signed rank test was used to compare untreated and colchicine-treated groups. Adjusted p-values are as follow: (C) N.T.: Not tested. FMF *p=0.016; (D) HD N.S. p=0.62, FMF *p=0.016.

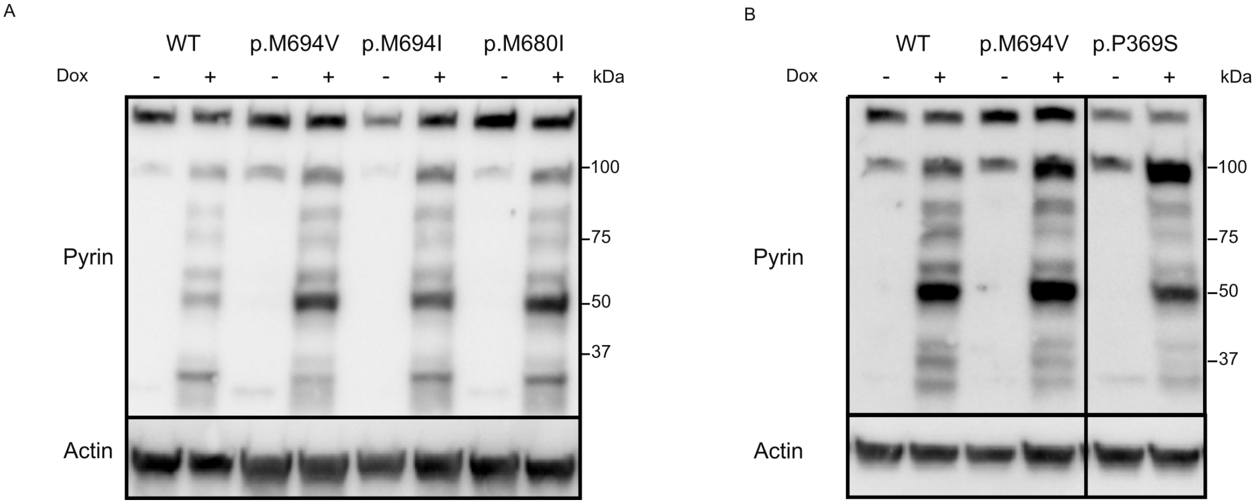

Figure S6. Doxycycline-mediated expression of p.M694I, p.M680I and p.P369S Pyrin variants.

(A-B) Expression of the indicated Pyrin variants was induced or not in U937 cells, as indicated, with doxycycline (Dox) and analyzed by Western blot. All the cell lines in panel B were tested on the same gel/ membrane (but not on adjacent lanes). Full Western blots are available in the Source Data.

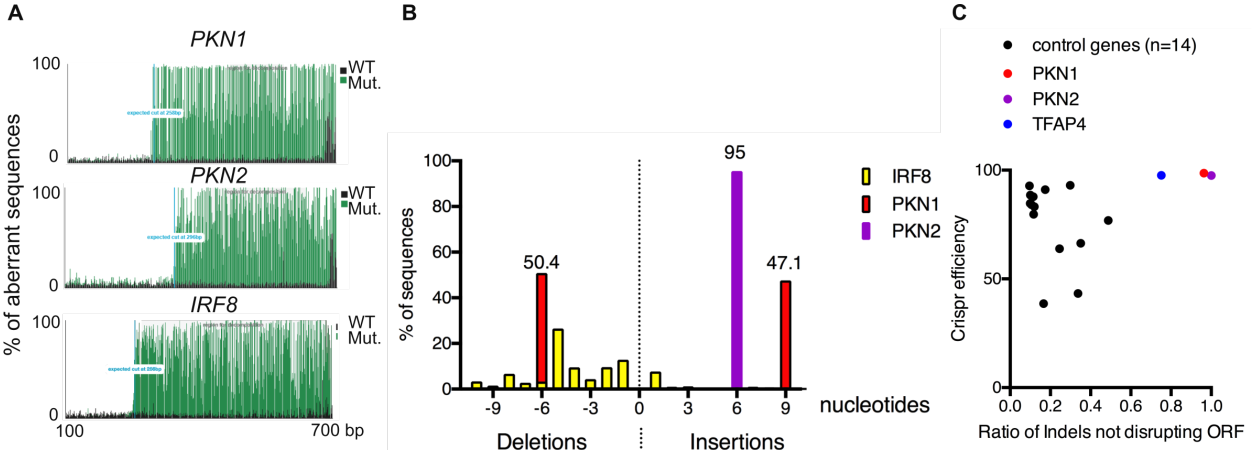

Figure S7. Edition of *PKN1/2* genes in U937 cells is associated with a strong bias towards indels not disrupting ORFs.

(A) Tide analysis of U937-Cas9 expressing cells targeted with sgRNA targeting both *PKN1* and *PKN2* (top two panels) or *IRF8* (lower panel) demonstrating efficient CRISPR/Cas9 editing at the sgRNA-targeted locus. (**B**) Tide analysis of the corresponding polyclonal cell populations demonstrating a strong bias towards indels not affecting the Open Reading Frames (ORFs) (i.e. -6 and +9 nt for *PKN1* and + 6nt for *PKN2*). Analysis of *IRF8*-edited population is shown as a control. One example out of three independent assays is shown. (**C**) Ratio of indels preserving the ORF in 17 independent cell lines generated by CRISPR/Cas9 at the same time as *PKN1/2*-edited U937 cells. *TFAP4* is known as a lethal target (Boboila *et al*, 2018) and is highlighted in blue as a control. Through clonal selection *PKN1*^{KO} were obtained, while we failed to obtain *PKN2*^{KO}.

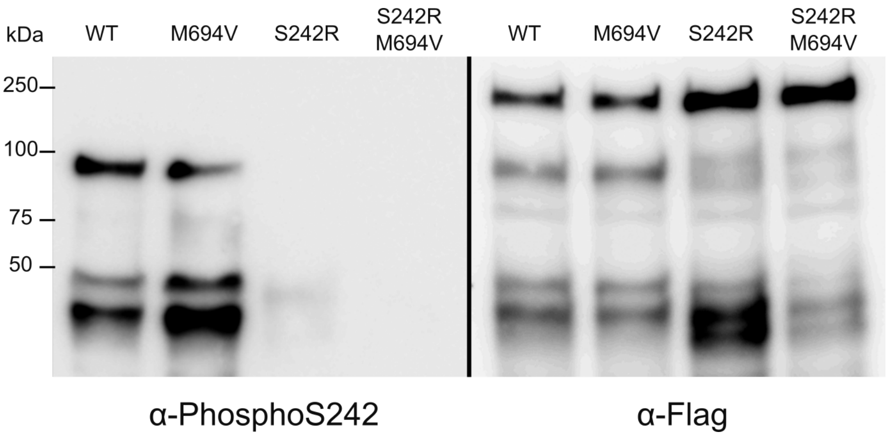

Figure S8. Validation of the anti-Phospho Pyrin antibody.

3xFLAG-Pyrin was immunoprecipitated in the cell lines expressing the indicated Pyrin variants. Western blot analysis using anti-phosphoS242 Pyrin and anti-Flag was performed. Note that the two degradation products around 50 kDa are heavily phosphorylated on S242 in both WT and p.M694V-expressing cells while the phosphorylation is undetectable in p.S242R Pyrin-expressing cells.

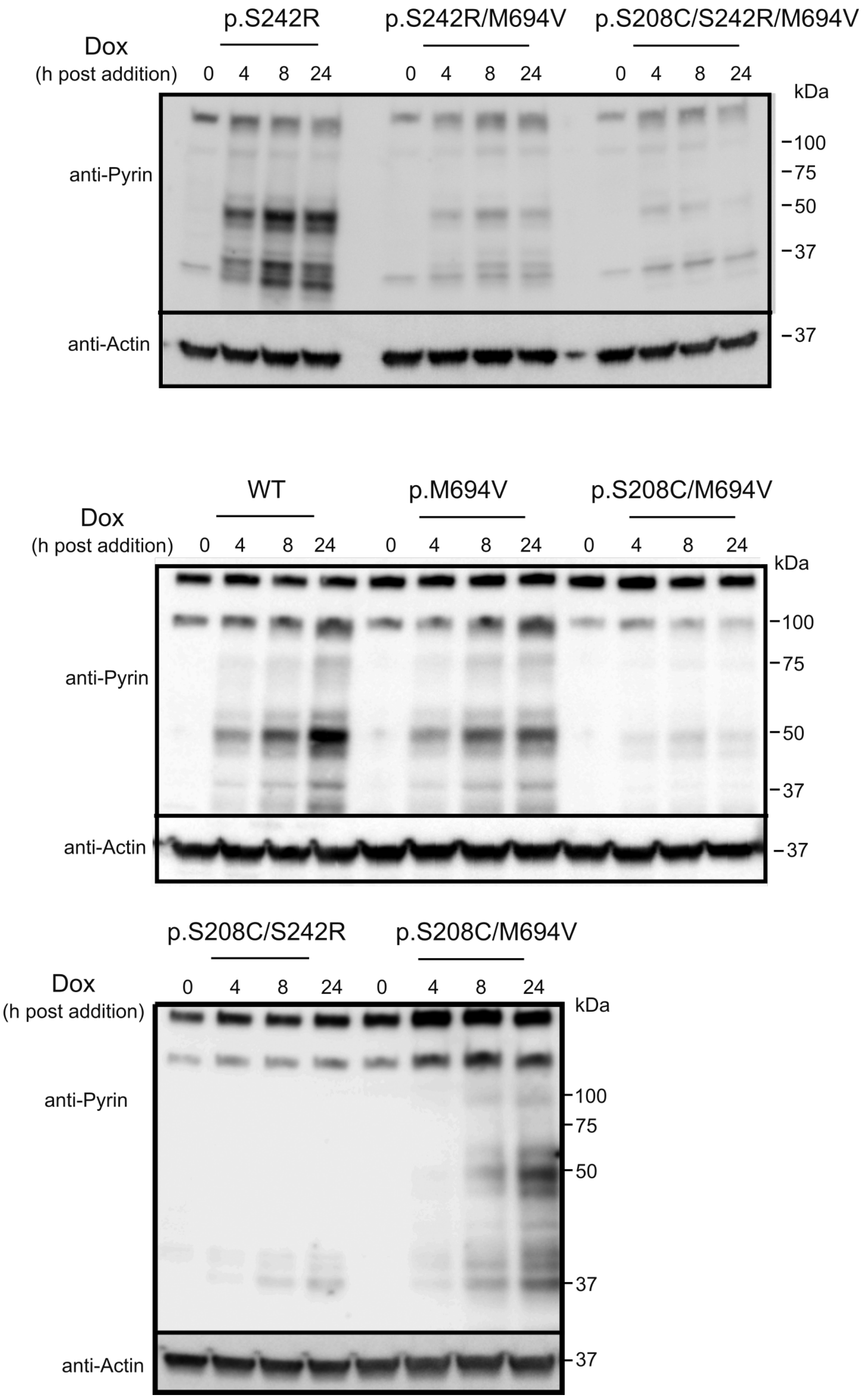

Figure S9. Doxycycline-mediated expression of p.S208C, p.S242R, p.S208C/S242R, p.S208C/M694V, p.S242R/M694V, p.S208C/S242R/M694V Pyrin variants.

Pyrin level was assessed by Western blot analysis in U937 cells expressing the indicated variants. All the cell lines express similar GFP level as WT Pyrin-expressing cells suggesting that the increased degradation is resulting from the specific mutations.

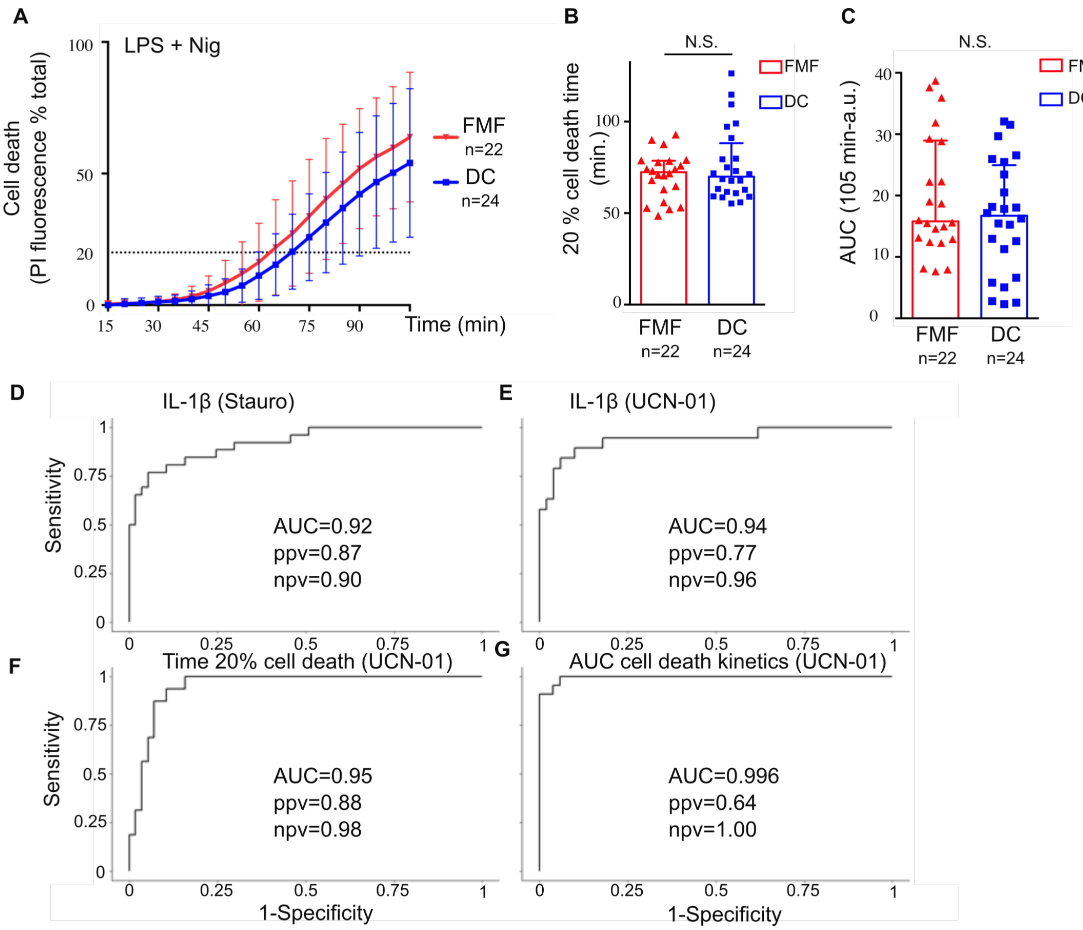

Figure S10. PKC inhibitors-mediated inflammasome activation specifically discriminates FMF patients from HD and from patients suffering from unrelated inflammatory conditions.

(A-C) Values from FMF patients are identical as the ones presented in Fig. 1. The figures were not merged for clarity issues.

(**A**) Monocytes from FMF patients or from unrelated disease controls (DC) were primed with LPS and treated with 5 μM nigericin (Nig). Cell death was monitored in real time by measuring propidium iodide (PI) influx/fluorescence every 5 minutes. (**B**) The time required to reach 20% cell death and (**C**) the area under the cell death kinetics curve (AUC) were computed for each patient. **(D-G)** Receiver Operating Characteristic (ROC) curves were computed for IL-1β concentration following (D) staurosporine or (E) UCN-01 treatment, (F) the time to obtain 20% cell death and (G) the area under the cell death kinetics curve. For each ROC curve, the AUC is indicated. An AUC of 1 indicates a perfect discrimination in terms of false negative (1-sensitivity) and false positive (specificity) between FMF patients and HD. The positive (ppv) and negative (npv) predictive values are indicated.

Data information

(A) Values were normalized based on the spontaneous and the total cell death values determined by Triton X100 treatment. (A) Each point of the curve corresponds to the average of the mean cell death values from three biological replicates of monocytes from the indicated number of DC or FMF patients. (B-C) Each symbol represents the value

from one patient. The bar represents the median ± interquartile range. (B) Not significant (N.S.) p=0.70 by Wilcoxon rank-sum test. (C) N.S. p=0.24 by unpaired t-test.

#	Sex	Age	Disease	MEFV Genotype	Colchicine	Symptoms at time of sampling
1	F	30	FMF	M694I/V726A	yes	yes
2	M	40	FMF	M694I/M694I	no	no
3	M	8	FMF	E148Q/M694V	yes	no
4	M	5	FMF	E167D,F479L; V377A	yes	no
5	M	13	FMF	E167D,F479L; V377A	yes	no
6	F	43	FMF	M694V/M694V	yes	no
7	M	30	FMF	M694I/M694I	yes	no
8	M	12	FMF	M694V/E148Q	yes	no
9	M	34	FMF	M694V/M694V	yes	no
10	M	5	FMF	M694V/M694V	yes	no
11	F	11	FMF	M694V/M694V	yes	no
12	F	8	FMF	M694V/R761H	yes	no
13	F	36	FMF	M694V/R761H	yes	no
14	M	16	FMF	M694I/M694I	yes	no
15	F	13	FMF	M694V/M694V	yes	no
16	F	9	FMF	M694V/M694V	yes	no
17	F	5	FMF	M694V/M694V	yes	no
18	F	37	FMF	M694I/M694I	yes	no
19	M	15	FMF	M694V/0	yes	no
20	F	8	FMF	M694V/M694V	no	no
21	F	10	FMF	M694V/M680I	yes	no
22	F	7	FMF	M694V/M694V	yes	no
23	F	15	FMF	M694V/M694V	yes	no
24	M	4	FMF	M694V/M694V	yes	no
25	F	17	FMF	M694V/M694V	yes	no
26	F	35	FMF	V726A/V726A	yes	no
27	F	8	FMF	M680I/V726A	yes	no
28	M	5	FMF	M694V/0	no	no
29	M	43	FMF	M694V/0	no	no
30	F	44	FMF	M694V/0	no	no
31	M	63	FMF	M694V/M694V	yes	no
32	M	9	FMF	M694V/M694V	yes	no
33	M	4	FMF	M694V/M694V	yes	no
34	M	41	FMF	M694I/0	yes	no
35	M	21	FMF	M694V/M694V	no	no
36	M	17	FMF	M694V/M694V	yes	no

37	F	44	FMF	M694V/M694V	yes	no
38	F	22	FMF	M680I/M694I	yes	no
39	F	32	FMF	M680I/M680I	yes	no
40	M	17	AOSD	NA	no	no
41	M	53	AOSD	NA	yes	no
42	F	53	AOSD	NA	no	no
43	F	32	Behçet	NA	yes	no
44	M	34	Behçet	NA	yes	no
45	F	28	Behçet	NA	yes	no
46	F	32	Behçet	NA	no	no
47	F	17	IBD	NA	no	no
48	F	17	IBD	NA	no	no
49	M	17	IBD	NA	no	no
50	M	13	IBD	NA	no	no
51	M	43	Lupus	NA	no	no
52	M	47	Lupus	NA	no	no
53	F	6	JIA	NA	no	no
54	F	28	sepsis	NA	no	no
55	F	27	sepsis	NA	no	no
56	M	89	sepsis	NA	no	no
57	F	13	sJIA	NA	no	no
58	F	16	sJIA	NA	no	no
59	M	3	MKD	NA	no	no
60	F	11	MKD	NA	no	no
61	M	5	MKD	NA	no	no
62	M	26	A20 deficiency	NA	no	no
63	F	4	H syndrome	NA	no	no
64	M	26	HD	NA	no	no
65	M	58	HD	NA	no	no
66	F	30	HD	NA	no	no
67	M	22	HD	NA	no	no
68	M	25	HD	NA	no	no
69	M	28	HD	NA	no	no
70	F	51	HD	NA	no	no
71	M	53	HD	NA	no	no
72	M	55	HD	NA	no	no
73	M	32	HD	NA	no	no
74	M	51	HD	NA	no	no
75	F	32	HD	NA	no	no
76	M	61	HD	NA	no	no
77	M	63	HD	NA	no	no
78	M	18	HD	NA	no	no
79	F	30	HD	NA	no	no

80	F	21	HD	NA	no	no
81	F	31	HD	NA	no	no
82	M	38	HD	NA	no	no
83	M	34	HD	NA	no	no
84	M	31	HD	NA	no	no
85	M	34	HD	NA	no	no
86	M	41	HD	NA	no	no
87	F	53	HD	NA	no	no
88	M	40	HD	NA	no	no
89	M	33	HD	NA	no	no
90	F	25	HD	NA	no	no
91	F	41	HD	NA	no	no
92	M	38	HD	NA	no	no
93	M	40	HD	NA	no	no
94	M	47	HD	NA	no	no
95	M	54	HD	NA	no	no
96	M	48	HD	NA	no	no
97	M	45	HD	NA	no	no
98	M	51	HD	NA	no	no
99	F	63	HD	NA	no	no
100	M	58	HD	NA	no	no
101	M	60	HD	NA	no	no
102	M	40	HD	NA	no	no
103	M	62	HD	NA	no	no
104	M	58	HD	NA	no	no
105	F	51	HD	NA	no	no
106	M	45	HD	NA	no	no
107	M	18	HD	NA	no	no
108	M	41	HD	NA	no	no
109	M	53	HD	NA	no	no
110	M	43	HD	NA	no	no

Table S1. Table of patients/ healthy donors (HD)

FMF: Familial Mediterranean Fever, AOSD: Adult Onset Still's Disease, IBD: Inflammatory Bowel Disease, JIA: Juvenile Idiopathic Arthritis, sJIA: Systemic juvenile Idiopathic Arthritis, MKD: Mevalonate Kinase Deficiency, NA: Not applicable.

Parameter	Inhib.	Threshold	Sens.	Spec.	PPV	NPV	Acc	AUC [Low-Up]
IL-1β	Stauro.	44	0.85	0.88	0.88	0.84	0.86	0.93 [0.86-0.99]
IL-1β	UCN-01	224	0.89	0.96	0.94	0.92	0.93	0.94 [0.86-1.00]
AUC_{RTCD}	UCN-01	21	0.94	1.00	1.00	0.96	0.98	0.98 [0.99-1.00]
$Time_{20\%CD}$	UCN-01	61	1.00	0.96	0.96	1.00	0.98	1.00 [0.99-1.00]

Table S2. Numerical parameters associated with the ROC curves presented in Fig. 5

The threshold values are indicated in pg/mL^{-1} for IL-1 β, in arbitrary units for the Area Under the real time cell death kinetics curves (AUC_{RTCD}) and minutes for the time to reach 20% cell death ($Time_{20\%CD}$). Sensitivity (Sens.), Specificity (Spec.), Positive Predictive Values (PPV), Negative Predictive Values (NPV), Accuracy (Acc.) for the indicated threshold values are shown. The Area under the ROC curve (AUC) are indicated with their lower and upper values calculated using a 95% confidence interval.

Site directed mutagenesis primers	
p.M694V-WT	5'-GGTACTCATTTTCCTTCATCATTATCACCACCCAGTAG-3'
	5'-CTACTGGGTGGTGATAATGATGAAGGAAAATGAGTACC-3'
WT-p.S242R	5'-GAAATGGTGACCTCAAGTCTTCTAGGTCGCATCTT-3'
	5'-AAGATGCGACCTAGAAGACTTGAGGTCACCATTTC-3'
WT-p.M680I	5'-TAAGCAGGAAAGGGAACATAACTCTGTCGCCAGAGAAT-3'<br>5'-ATTCTCTGGCGACAGAGTTATGTTCCCTTTCCTGCTTA-3'
WT-p.M694I	5'-ACTGGGTGGTGATAATGATAAAGGAAAATGAGTACCAG-3'<br>5'-CTGGTACTCATTTTCCTTTATCATTATCACCACCCAGT-3'
WT-p.P639S	5'-GGAAGCCTAAGCTCCCAGCCCCTGC-3'<br>5'-GCAGGGGCTGGGAGCTTAGGCTTCC-3'
WT-p.S208C	5'-CCCCGCGGAGCAGGCGTTTCTGC-3'<br>5'-GCAGAAACGCCTGCTCCGCGGGG-3'
**sgRNA**	
PKN1 sgRNA1	5'-CGGGGCCTTTACAGCCGAAG-3'
PKN1 sgRNA2	5'-ACCCTGGAGGTACGAGTGGT-3'
PKN2 sgRNA1	5'-GAGGGAGCGACTAGACGAAC-3'
PKN2 sgRNA2	5'-ACCCCGAACGGGGGGAGATT-3'
**siRNA**	
NT	# 4390843 (ThermoFischer)
PKN2 siRNA#12	5'-GAUAUCAAGGAUCGAAUUA-3'
PKN2 siRNA#13	5'-GAAUGUGAGUGCUGUUCAA-3'
PKN2 siRNA#14	5'-GGAGCGCUCUGAUGGACAA-3'

Table S3. Primer, sgRNA and siRNA table

NT: Non-targeting
