# Supplementary material for: Pyrin dephosphorylation is sufficient to trigger inflammasome activation in familial Mediterranean fever patients
Source: EMBO Mol Med. 2019 Oct 7;11(11):e10547. doi: 10.15252/emmm.201910547 (PMC6835204; doi:10.15252/emmm.201910547)
Supplement: Supplementary file 2 — Expanded View Figures PDF [file EMMM-11-e10547-s002.pdf]

Expanded View Figures

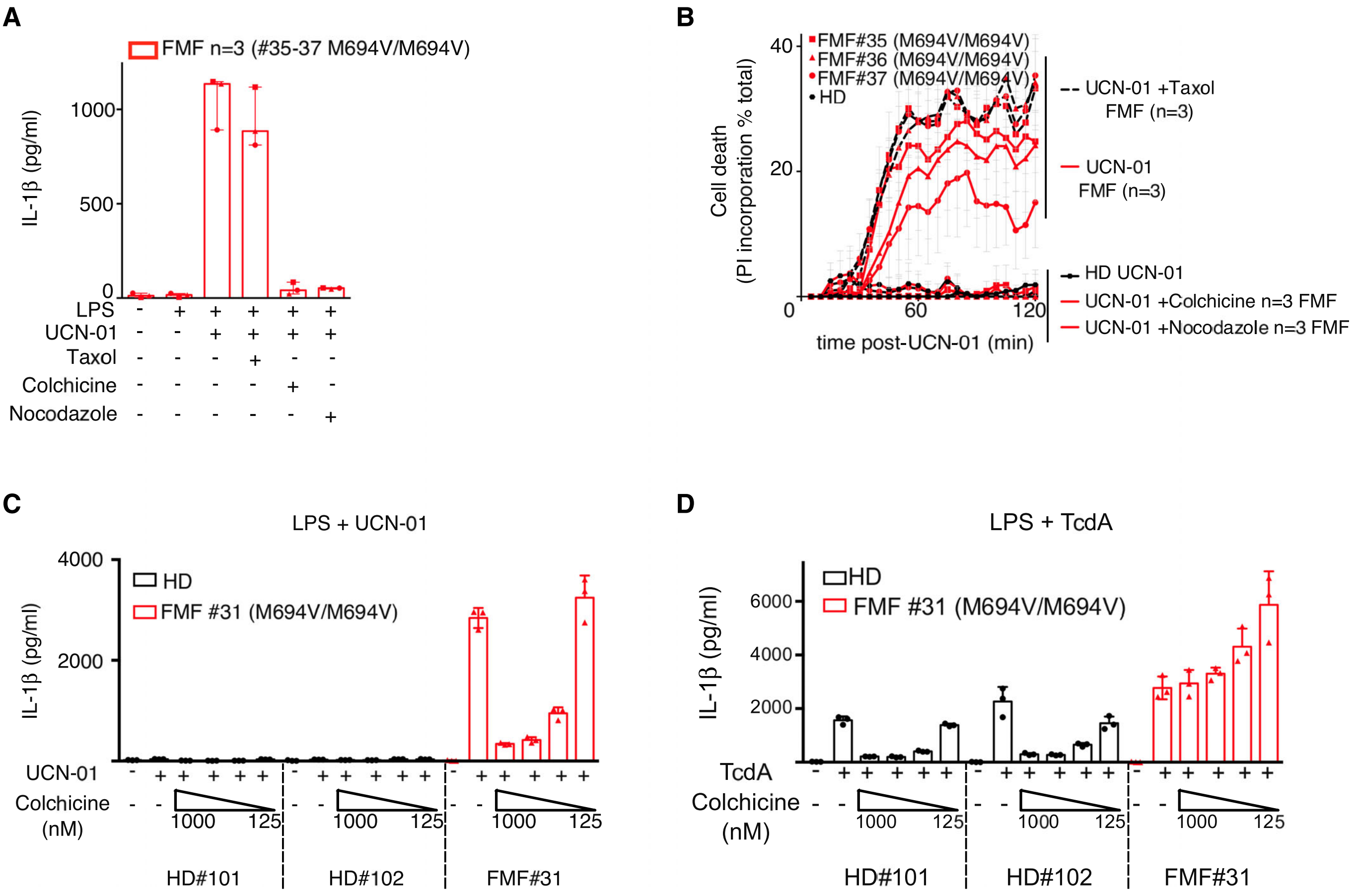

Figure EV1. Nocodazole and colchicine, the latter in a dose-dependent manner, inhibit UCN-01-mediated responses.

A Primary human monocytes from FMF patient were primed with LPS and stimulated as indicated with UCN-01 in the presence of paclitaxel (Taxol, 5 μM), nocodazole (5 μM), or colchicine (1 μM).

B Propidium iodide incorporation was monitored every 5 min post-UCN-01 addition in the presence of Taxol (5 μM), nocodazole (5 μM), or colchicine (1 μM). PI incorporation was normalized using TX-100 cells (total PI incorporation). (A, C, D) IL-1β concentration in the supernatant was quantified by ELISA.

C, D Primary human monocytes from the indicated healthy donor (HD) or FMF patient were primed with LPS and stimulated as indicated with (C) UCN-01 or (D) TcdA (1 μg/ml) in the presence of the indicated concentration of colchicine.

Data information: (A) Each symbol corresponds to the mean of a biological triplicate for one FMF patient (square, triangle, and round, patients #35, 36, 37 (all M694V/M694V), respectively), and the bar shows the median ± interquartile range. (B) Each symbol represents the mean (± SD) of a biological triplicate for one FMF patient. (C, D) Each dot represents one biological replicate, and the bar shows the mean of a biological triplicate from one individual.

© 2019 The Authors

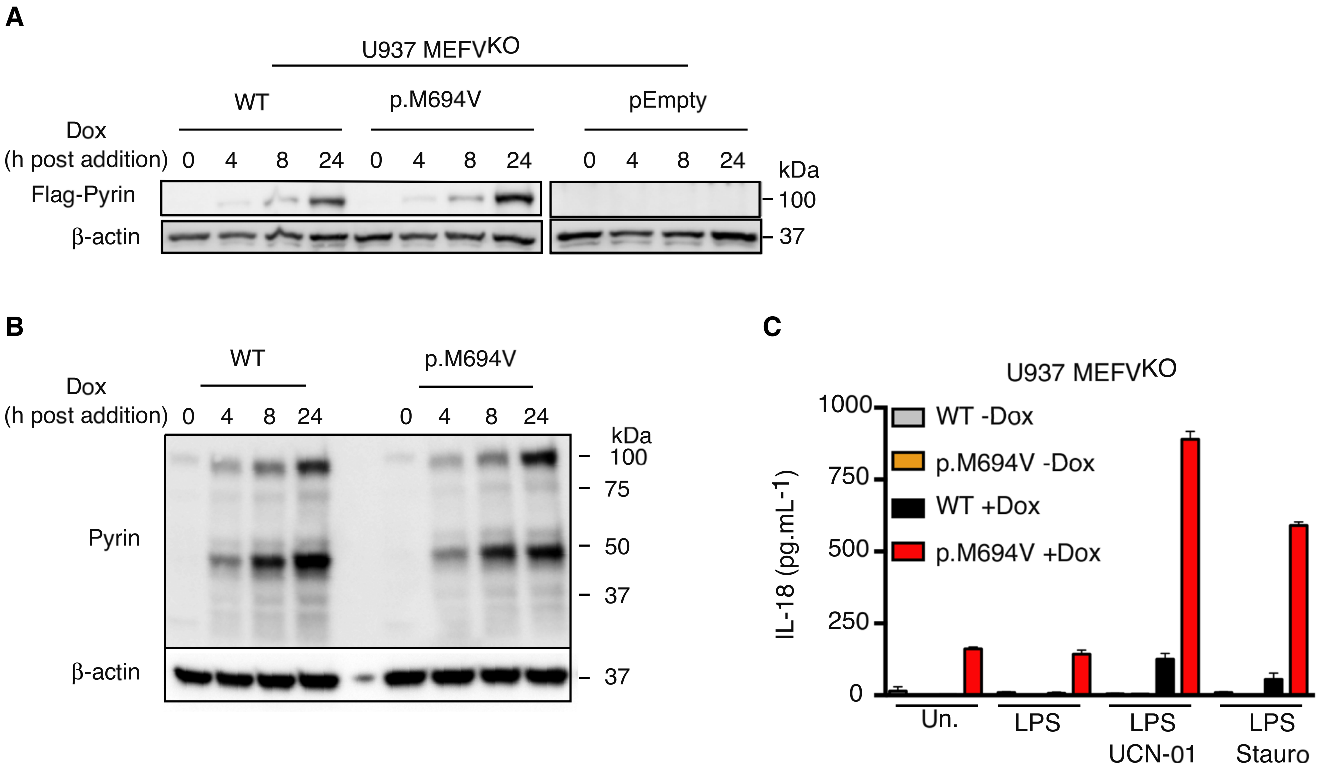

Figure EV2. Doxycycline-mediated expression of p.[M694V] *MEFV* is necessary and sufficient to confer to PKC inhibitors the ability to trigger IL-18 release.

U937 MEFV^{KO} cells expressing the indicated plasmids were treated, when indicated, with doxycycline for the indicated time

A, B The expression of Flag-Pyrin was revealed by Western blotting analysis against Flag (A) or against Pyrin (B).

C IL-18 levels were quantified in the supernatant of PMA-differentiated U937 cells by ELISA at 3 h post-treatment with UCN-01 or staurosporine (Stauro). One experiment representative of two independent experiments is shown. The bar represents the mean of a biological triplicate.

Source data are available online for this figure.

 © 2019 The Authors

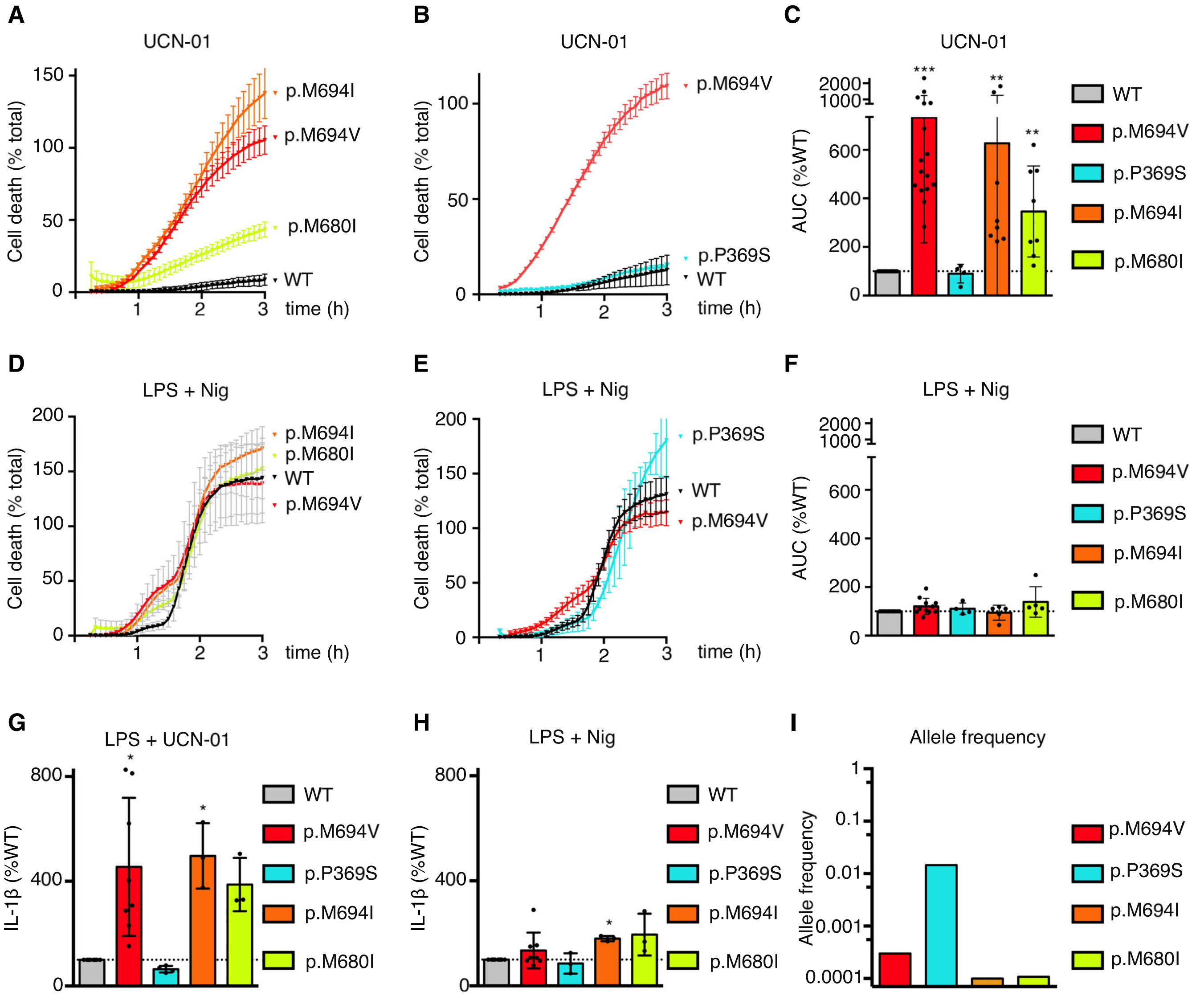

Figure EV3. Expression of the p.M694I or the p.M680I, but not of the p.P369S, Pyrin variants triggers inflammasome activation in response to UCN-01.

The expression of the indicated Pyrin variants was induced in U937 cells using doxycycline (Appendix Fig S6).

A–F Propidium iodide (PI) influx/fluorescence was monitored every 5 min for 3 h in response to UCN-01 (A–C) or LPS + nigericin (D–F).
G, H U937 cells were differentiated in macrophages using PMA, treated with LPS and UCN-01 (G) or nigericin (H). IL-1β levels were quantified by ELISA.
I The frequency of the different *MEFV* alleles is shown (data extracted from gnomad).

Data information: Cell death was normalized using PI incorporation in TX-100-treated cells. Panels (A, B, D and E) result from two independent transduction experiments and are compared with their respective control cells. One experiment representative of 4 (B, E), 5 (D) to 8 (A) independent experiments is shown. Mean and standard deviations from three biological replicates are shown. (C, F) Area under the curve (AUC) was normalized to WT-treated cells. One dot represents the average of a biological triplicate, and the bar represents the mean ± SD of 4–8 independent experiments as indicated. (G, H) IL-1β concentrations were normalized to WT-treated cell levels. One dot represents the average of a biological triplicate, and the bar represents the mean ± SD of three independent experiments. (C, F, G, H) The horizontal dotted line corresponds to the value of U937 cells expressing WT Pyrin (normalized to 100%). (C, F, G, H) Values did not pass the D'Agostino and Pearson omnibus normality test; Kruskal–Wallis analysis with Dunn's correction for multiple comparisons was applied, and two-tailed *P*-values are shown. (C) WT vs. M694V ****P* < 0.0001; WT vs. M694I ***P* = 0.0011; WT vs. M680I ***P* = 0.0065 (G) WT vs. M694V **P* = 0.0214; WT vs. M694I **P* = 0.0476 (H) WT vs. M694I **P* = 0.0431.

© 2019 The Authors

Figure EV4. SiRNA-mediated knock-down of *PKN2* in *PKN1*^{KO} cells expressing p.M694V Pyrin increases cell death.

A PKN1^{KO} clones expressing p.M694V or WT Pyrin were obtained and confirmed by Western blot analysis. Four clones are shown. Clones 3 and 6 were selected.

B Absolute transcript level of the indicated genes was quantified by RNAseq in U937 cells (Benaoudia *et al*, 2019) and expressed as count per million (cpm). *PYCARD* encodes ASC and is shown as a reference.

C *PKN2* transcript levels were assessed in U937 cells at 24 h post-electroporation with the indicated non-targeting (NT) siRNA or three different siRNAs targeting *PKN2*.

D–F U937 cells with the indicated Pyrin variant were treated (+Dox, plain lines) or not (No Dox, dotted lines) with doxycycline (Dox) at 24 h post-electroporation with the indicated siRNA. Cell death was monitored every 15 min. (F) SiRNA 14 did not reduce substantially *PKN2* levels.

Data information: (D, E, F) Cell death was normalized to the maximal propidium iodide (PI) incorporation determined using TX-100-treated cells. Each dot represents the mean ± SD of a biological triplicate from one experiment representative of three independent experiments.

Source data are available online for this figure.

© 2019 The Authors

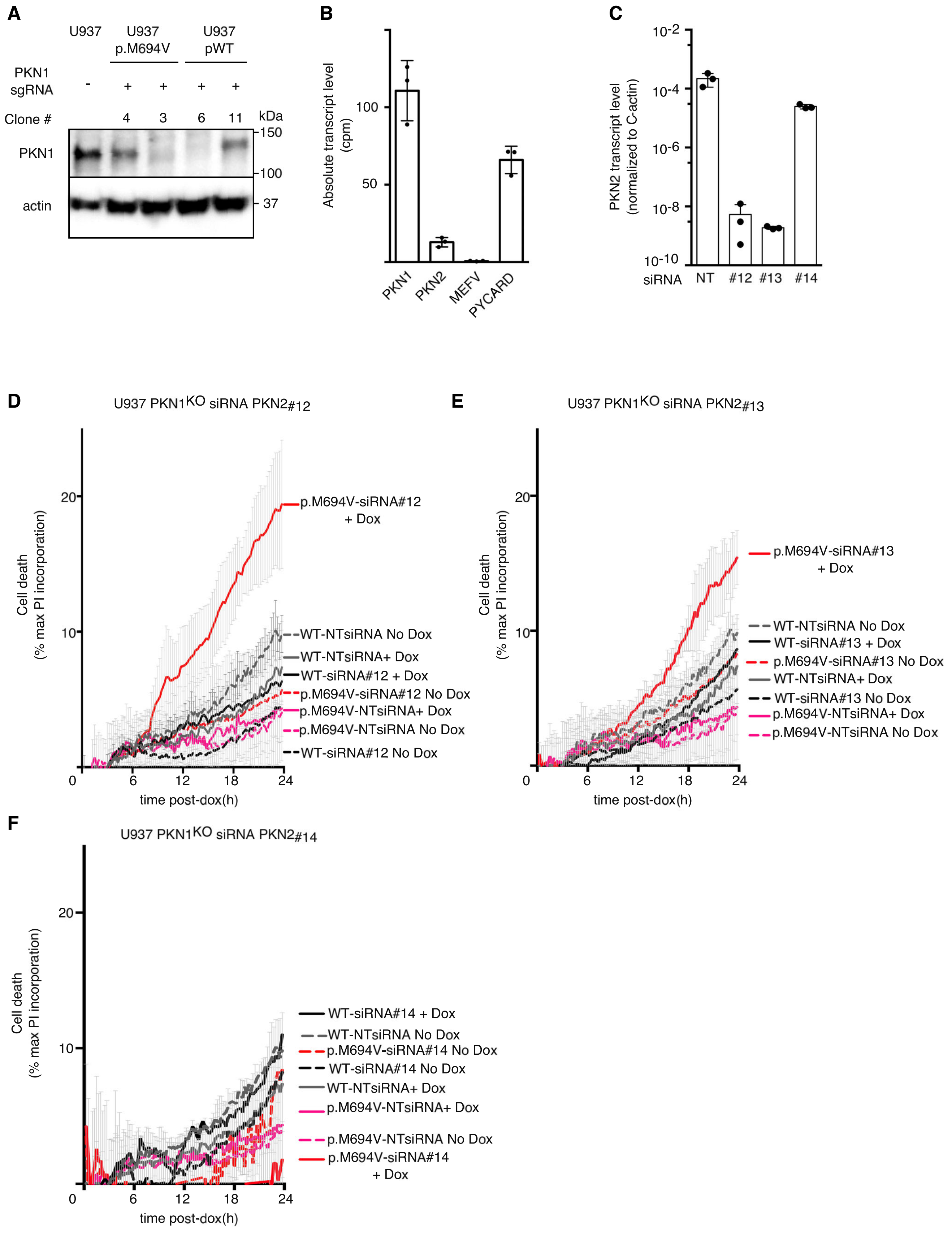
A
U937
U937 p.M694V
U937 pWT
PKN1 sgRNA
Clone #
4 3 6 11
kDa
150
100
37
PKN1
actin
B
Absolute transcript level (cpm)
PKN1
PKN2
MEFV
PYCARD
C
PKN2 transcript level (normalized to C-actin)
siRNA
NT #12 #13 #14
D
U937 PKN1KO siRNA PKN2#12
Cell death (% max PI incorporation)
time post-dox(h)
p.M694V-siRNA#12 + Dox
WT-NTsiRNA No Dox
WT-NTsiRNA+ Dox
WT-siRNA#12 + Dox
p.M694V-siRNA#12 No Dox
p.M694V-NTsiRNA+ Dox
p.M694V-NTsiRNA No Dox
WT-siRNA#12 No Dox
E
U937 PKN1KO siRNA PKN2#13
p.M694V-siRNA#13 + Dox
WT-NTsiRNA No Dox
WT-siRNA#13 + Dox
p.M694V-siRNA#13 No Dox
WT-NTsiRNA+ Dox
WT-siRNA#13 No Dox
p.M694V-NTsiRNA+ Dox
p.M694V-NTsiRNA No Dox
F
U937 PKN1KO siRNA PKN2#14
WT-siRNA#14 + Dox
WT-NTsiRNA No Dox
p.M694V-siRNA#14 No Dox
WT-siRNA#14 No Dox
WT-NTsiRNA+ Dox
p.M694V-NTsiRNA+ Dox
p.M694V-NTsiRNA No Dox
p.M694V-siRNA#14 + Dox

Figure EV4.

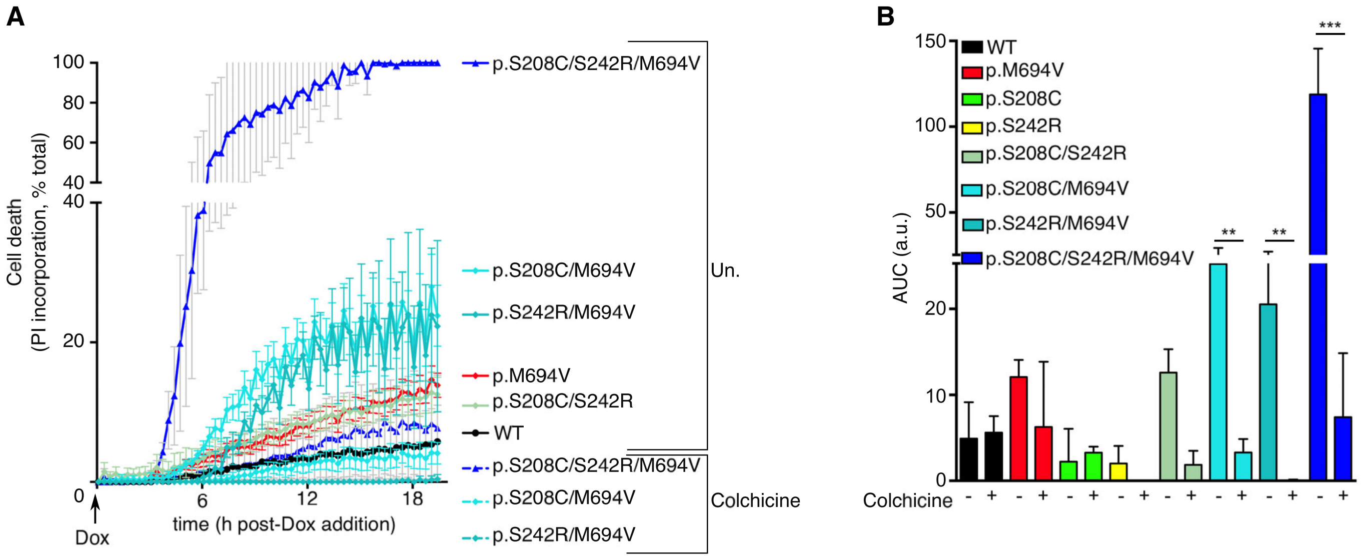

Figure EV5. Colchicine blocks cell death mediated by the expression of p.S208C/M694V, p.S242R/M694V, or p.S208C/S242R/M694V variants.

U937 cells bearing the indicated plasmids were treated at time 0 with doxycycline (Dox) in the presence or not as indicated of colchicine.

A Propidium iodide (PI) influx/fluorescence was monitored every 15 min for 20 h.

B Area under the curve (AUC) corresponding to Fig EV5A is shown.

Data information: (A) Cell death was normalized using TX-100-treated cells. Each dot represents the mean ± SD of a biological triplicate from one experiment representative of three independent experiments. (B) The bar represents the mean ± SD of a biological triplicate from one experiment representative of three independent experiments. One-way ANOVA with Sidak's multiple comparison test was performed to assess the effect of colchicine in each cell line. *P*-values from left to right: ***P* = 0.0011; ***P* = 0.0079; ****P* < 0.0001.

 © 2019 The Authors
