# Supplementary figures and images for: Pyrin dephosphorylation is sufficient to trigger inflammasome activation in familial Mediterranean fever patients
Source: EMBO Mol Med. 2019 Oct 7;11(11):e10547. doi: 10.15252/emmm.201910547 (PMC6835204; doi:10.15252/emmm.201910547)

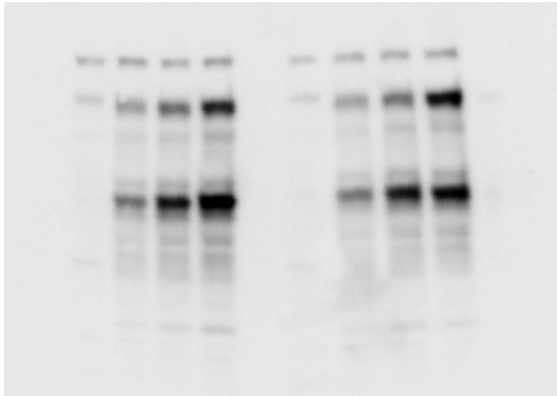

Anti-Pyrin

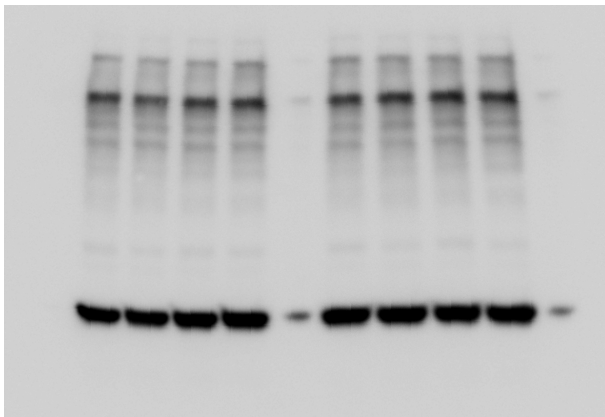

Anti-Actin

Supplement: Supplementary file 3 — Source Data for Expanded View and Appendix [file EMMM-11-e10547-s009.zip › EV_appendix_source_data/FigEV2B-SourceData.pdf]

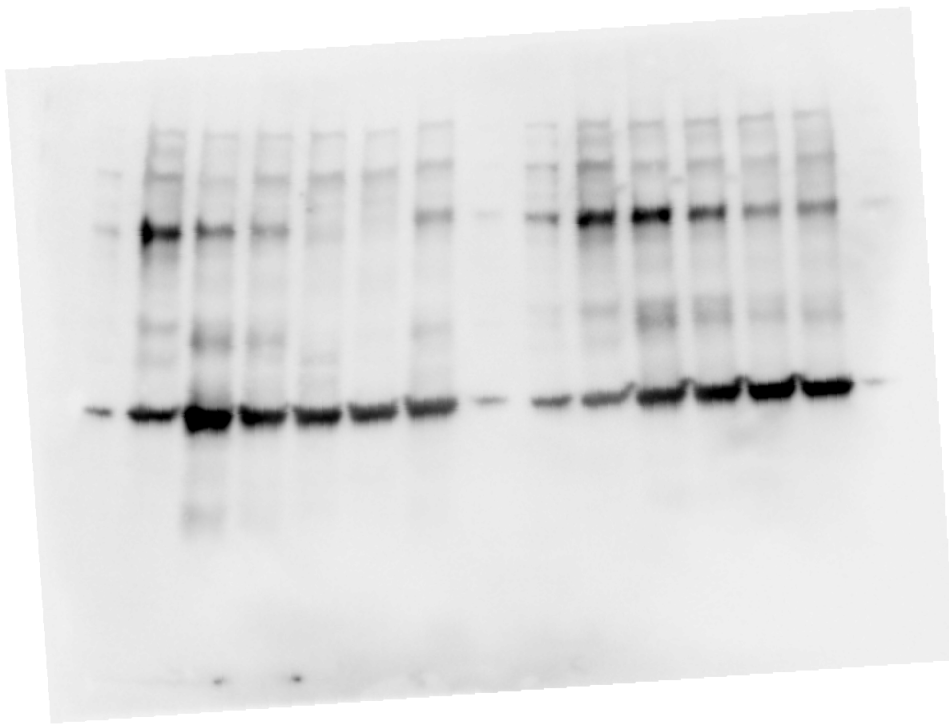

anti-PKN1

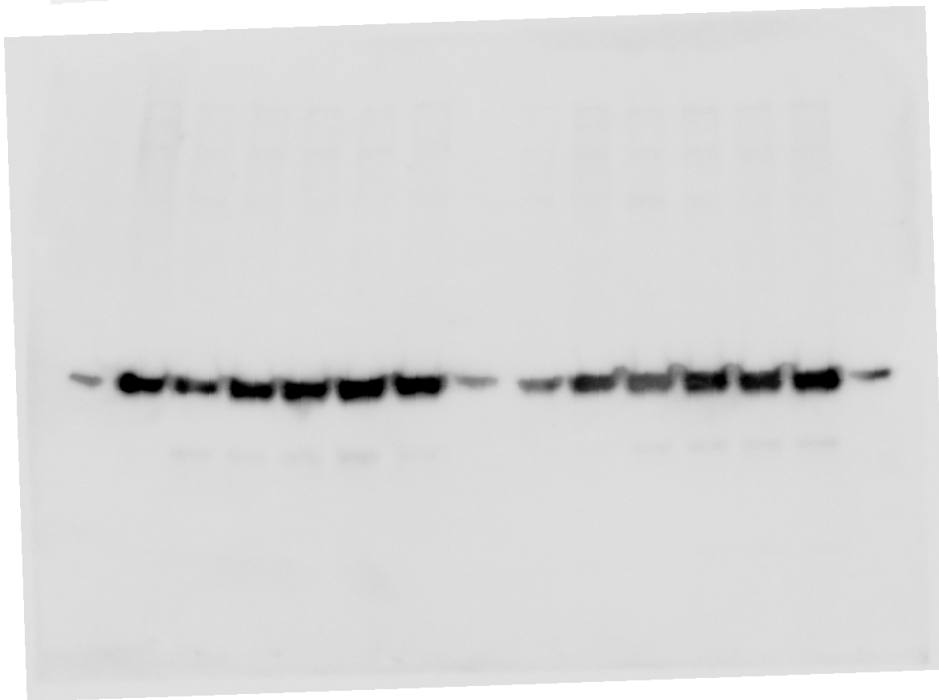

anti-Actin

Supplement: Supplementary file 3 — Source Data for Expanded View and Appendix [file EMMM-11-e10547-s009.zip › EV_appendix_source_data/FigEV4_sourceData.pdf]

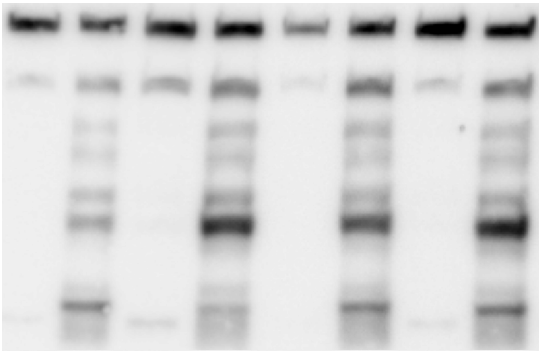

S5A anti Pyrin

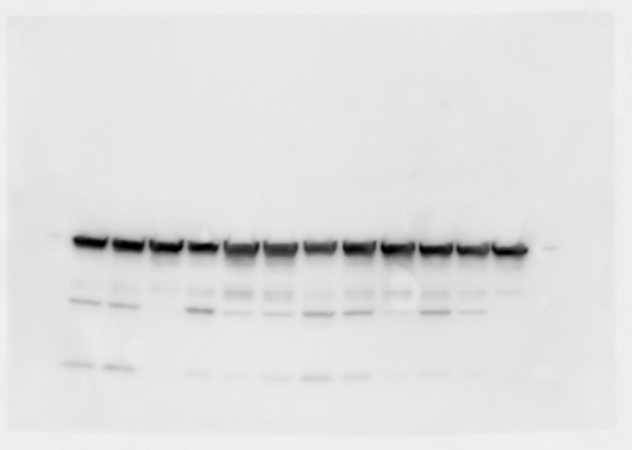

S5A anti-Actin

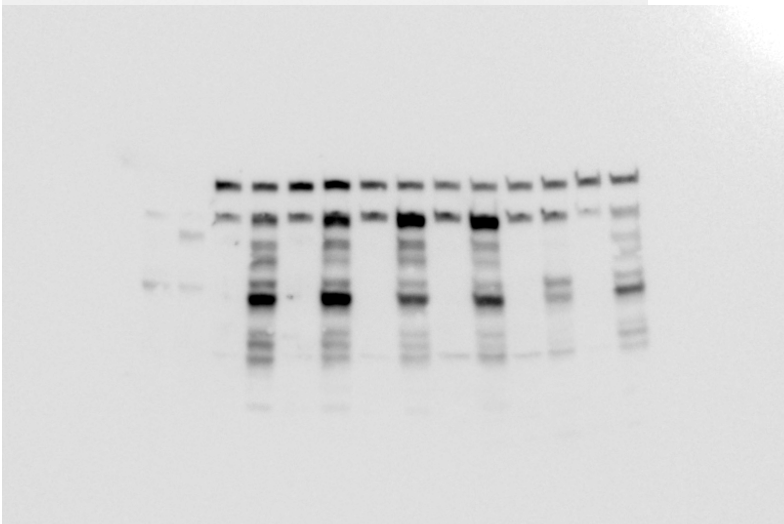

S5B anti Pyrin

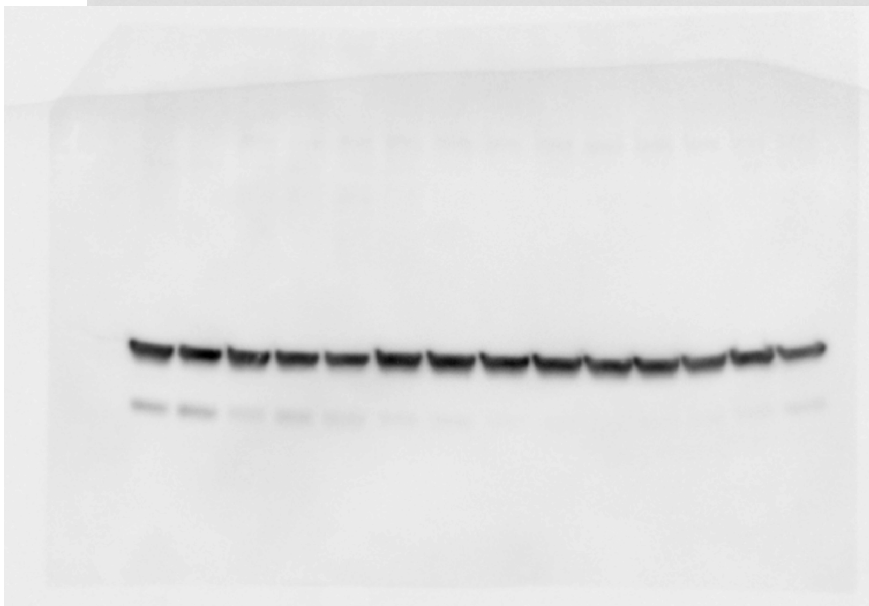

S5B anti Actin

Supplement: Supplementary file 3 — Source Data for Expanded View and Appendix [file EMMM-11-e10547-s009.zip › EV_appendix_source_data/FigS6_SourceData.pdf]

A

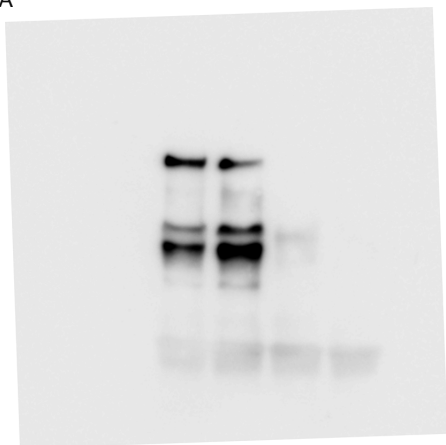

B

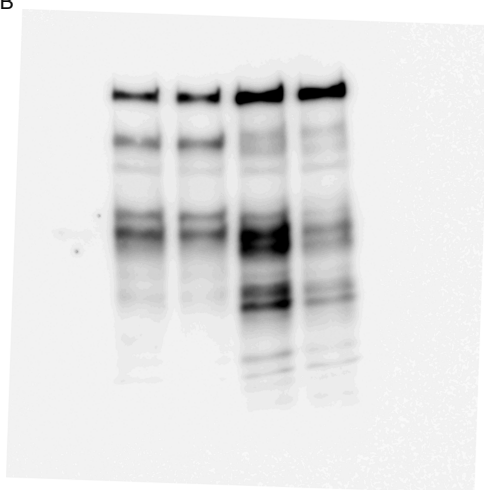

Supplement: Supplementary file 3 — Source Data for Expanded View and Appendix [file EMMM-11-e10547-s009.zip › EV_appendix_source_data/FigS8_SourceData_FullWB-coIP-PhosphoBlotsvg.pdf]

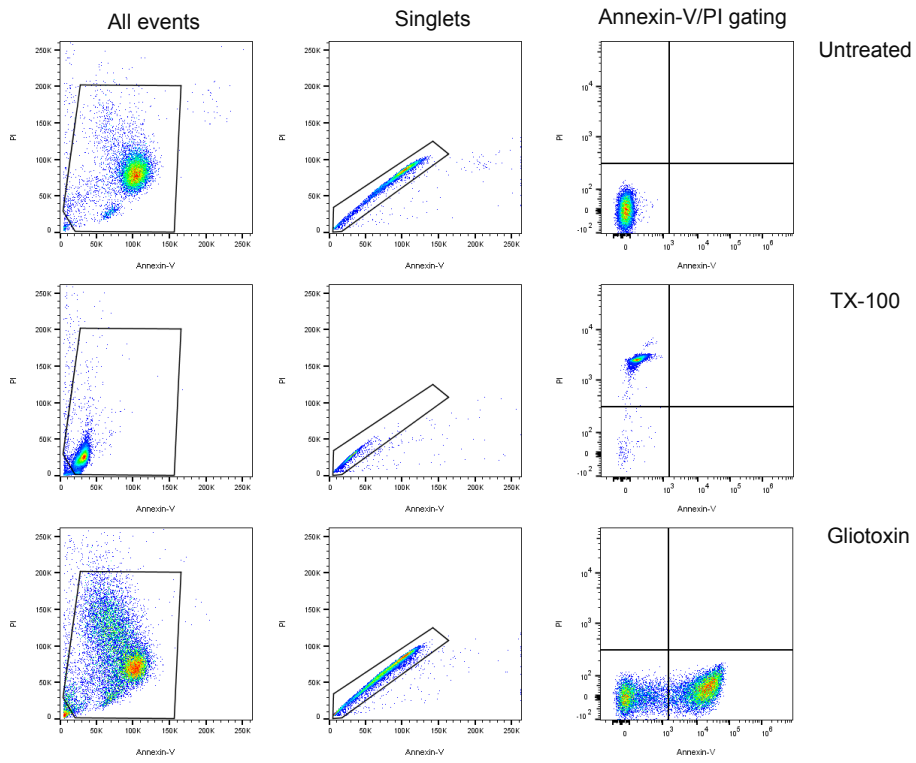

Controls and gating strategy for Fig 2D

Supplement: Supplementary file 6 — Source Data for Figure 2 [file EMMM-11-e10547-s004.zip › emmm201910547-sup-0005-SDataFig2.pdf]

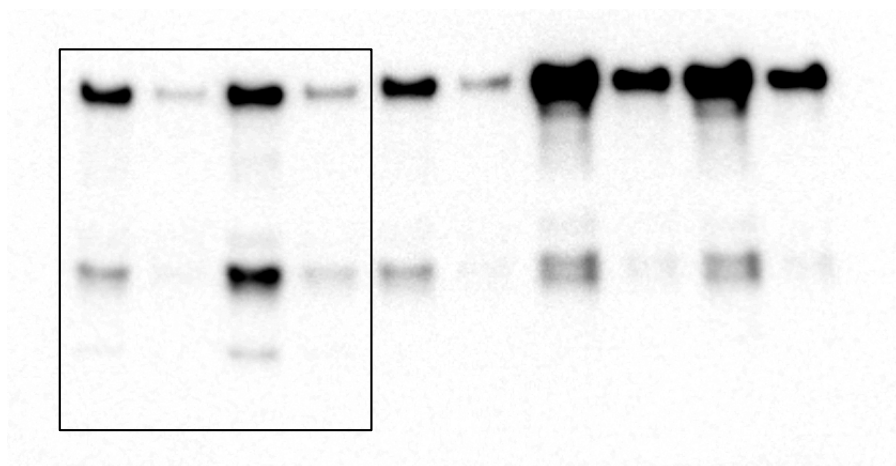

PhosphoS242  
Pyrin

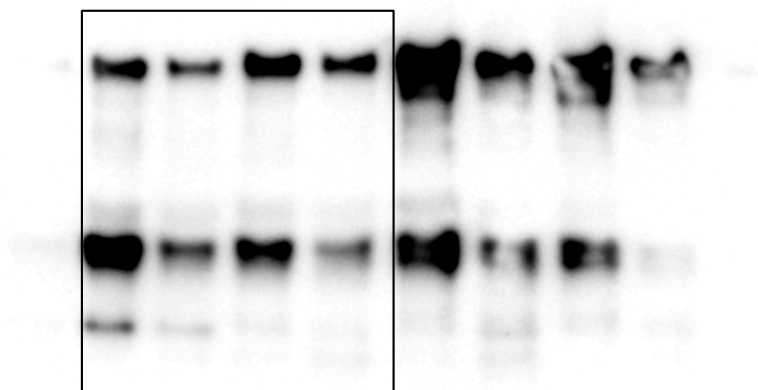

Pyrin

Supplement: Supplementary file 8 — Source Data for Figure 4 [file EMMM-11-e10547-s006.zip › emmm201910547-sup-0008-SDataFig4.pdf]
